# Free convection in a square wavy porous cavity with partly magnetic field: a numerical investigation

**DOI:** 10.1038/s41598-024-64850-7

**Published:** 2024-06-19

**Authors:** Amirmohammad Mirzaei, Bahram Jalili, Payam Jalili, Davood Domiri Ganji

**Affiliations:** 1 Department of Mechanical Engineering, Sharif University of Technology, Tehran, Iran; 2 Department of Mechanical Engineering, North Tehran Branch, Islamic Azad University, Tehran, Iran; 3 Department of Mechanical Engineering, Babol Noshirvani University of Technology, P.O. Box 484, Babol, Iran

**Keywords:** Partial magnetic field, Natural convection, Porous medium, Square wavy cavity, Mechanical engineering, Fluid dynamics

## Abstract

Natural convection in a square porous cavity with a partial magnetic field is investigated in this work. The magnetic field enters a part of the left wall horizontally. The horizontal walls of the cavity are thermally insulated. The wave vertical wall on the right side is at a low temperature, while the left wall is at a high temperature. The Brinkman-Forchheimer-extended Darcy equation of motion is utilized in the construction of the fluid flow model for the porous media. The Finite Element Method (FEM) was used to solve the problem’s governing equations, and the current study was validated by comparing it to earlier research. On streamlines, isotherms, and Nusselt numbers, changes in the partial magnetic field length, Hartmann number, Rayleigh number, Darcy number, and number of wall waves have been examined. This paper will show that the magnetic field negatively impacts heat transmission. This suggests that the magnetic field can control heat transfer and fluid movement. Additionally, it was shown that heat transfer improved when the number of wall waves increased.

## Introduction

Natural convection is vital in various engineering applications, including heat transfer in electronic devices, energy-efficient building design, and thermal management in industrial processes. To design and optimize such systems, it is important to understand and predict the fluid flow and heat transfer properties in complex geometries^1–6^.

Several studies have focused on the influences of magnetic fields on heat transfer and fluid flow in square wavy cavities. Researchers have examined the influence of different parameters, for instance, magnetic field strength, orientation, and boundary conditions, on the convective heat transfer process. Sreedevi et al.^7^ examined the behavior of nanofluid in a square cavity considering magnetic field and thermal radiation. The finite difference approach was used to solve the governing equations. They found that the maximum average value of the Nusselt number can be obtained with higher values of the Rayleigh number. To study the natural convection in a cavity where an inclined oval heater is exposed to a magnetic field, Dogonchi et al.^8^ performed a numerical investigation using the CVFEM method. According to their findings, improving the Hartmann number reduced the average Nusselt at a constant Rayleigh number. Li et al.’s research^9^ concentrated on convective heat transfer in an alumina water nanofluid-filled square cavity that was angled toward the horizon. In addition to convective heat transfer, they also considered radiative heat transfer. The study also analyzed entropy production. A hot circular baffle was placed inside the cavity under a fixed horizontal magnetic field. They understood that with the increase of the Rayleigh number from 103 to 106, the pace of heat transfer improved 4.5 times, and the entropy produced also increased with the development of the Rayleigh number. Furthermore, as the magnetic field strength (Ha) increased, there was a notable decrease in both the average Nusselt number (Nu) and the amount of entropy generated. Specifically, the Nu decreased by 45%, while the generated entropy diminished by 35%. In order to study the entropy generation and heat transfer in a porous corrugated cavity filled with an integrated nanofluid under the influence of natural convection, Dogonchi et al.^10^ conducted a numerical study using the finite element method (FEM). This study shows that raising the Rayleigh number (Ra), and Darcy number (Da) is necessary to raise the thermal-natural convection rate. The results showed that complete irreversibility is established with Ra and Da, while it is reduced with Ha. Also, this analysis provides valuable insights into entropy production and convective heat transfer characteristics in such complex systems. Jalili et al. ^11–17^ did several works using the FEM method in this field. Mahmoudi ^18^ conducted theoretical and numerical research to investigate natural convection heat transfer in a porous cavity exposed to a magnetic field. This study used the scale analysis method to create a correlation to predict the heat transfer. A numerical study was also conducted using the Lattice Boltzmann method to validate the theoretical findings. The numerical simulations’ results were consistent with the theoretical predictions, indicating the proposed correlation’s correctness and reliability. This research contributes to a better understanding of heat transfer in porous cavities with magnetic field effects. Izadi et al. ^19^ examined the influence of two non-constant magnetic sources on the natural convection of a magnetic nanofluid in a porous medium. They discovered that as the intensity ratio between the two magnetic sources grew along with a rise in the Rayleigh (Ra) numbers, the Nusselt number initially dropped. This suggests that the convection rate decreases when the magnetic field strength becomes more asymmetric. It highlights the complex interaction between magnetic fields and natural convection in porous media and provides further insights into the behavior of such systems. Massoudi et al. ^20^ studied the physical properties of natural convection and radiation heat transfer in a nonagon cavity with a variable magnetic field length in a porous medium with nanofluid exposed to a uniform magnetic field. In order to find out how metal foam and rotation angle affected the natural convection of nanofluids in a hollow exposed to a magnetic field, Qi et al. ^21^ conducted an experiment. Their research demonstrated that a horizontal magnetic field lessens the cavity’s heat transfer, but a vertical direction improves it. The horizontal magnetic field with more force brings down the number of Nusselt, and the vertical magnetic field with higher strength enhances heat transfer, resulting in a larger Nusselt number. The numerical examination of natural convection in an F-shaped cavity with a horizontal periodic magnetic field containing non-Newtonian silver nanofluid in a porous medium was conducted by AK Hussein et al. ^22^. It was noticed that heat transfer improves with increasing solid volume fraction (φ), Darcy number (Da), and Rayleigh number (Ra). Izadi et al. ^23^ numerically studied nanofluid’s natural convection in a porous medium influenced by a nonuniform magnetic field. Buoyancy forces, Lorentz forces, and magnetism influence the hybrid nanofluid. They found that in a porous medium at high Da, the Nusselt number falls as the porosity coefficient rises, but the Nusselt number is unaffected by the porosity coefficient of the porous medium at low and high values of Da and Ra, respectively. At low Rayleigh numbers, with the change of porosity and permeability coefficient, natural convection heat transfer change is not noticeable. Hashemi et al. ^24^ studied the natural convection of micropolar copper–water nanofluid in a porous chamber that produces heat. Their investigation focused on the thermal and dynamic characteristics of the nanofluid in a square chamber, where heat is produced in both the fluid and solid phases of the porous medium. They employed the Galerkin finite element method with a nonuniform structured grid to solve the governing equations and the Darcy model to simulate the flow dynamics. Finally, they showed the effect of various dimensionless parameters on velocity, temperature, and rotation. Fenghua Li et al. ^25^ explored the flow behavior of hybrid nanomaterials inside a permeable cavity under the influence of magnetic force. They considered the effects of permeability and external force in the Navier–Stokes equations to simulate the free convection of hybrid nanomaterials. They also evaluated the effect of various factors such as Darcy number, Hartmann number, and radiation on the fluid flow. The steady-state flow of magnetized nanofluid in the wavy cavity with radiation was examined by Nong et al. ^26^. The control volume finite element (CVFE) technique is used to estimate numerical modeling. The behavior of streamlines, average Nusselt number, and isotherms was shown to be affected by magnetic force, Rayleigh number, porosity coefficient, and shape factors of nanoparticles according to them. The natural convection of a micropolar fluid was examined by Nikita et al. ^27^. They reviewed their study in the wave cavity. They discussed how the flow patterns, temperature fields, and average Nusselt number in the hot corrugated wall were affected by various characteristics, including the Rayleigh number, Prandtl number, wave number, and vortex viscosity parameter. Their work is based on partial differential equations constructed in non-dimensional variables and solved with second-order precision using a finite difference approach. Mahmoud et al. ^28^ focused on investigating fluid flow and heat transfer in a porous media when subjected to a magnetic field. They investigated the effect of different parameters such as Darcy number, porosity parameter, radiation parameter, and Richardson number on heat transfer and flow characteristics. They applied numerical techniques and the Galerkin weighted residual finite element approach to solve the governing equations. According to their findings, there is a positive correlation between these factors and heat transfer, while an increase in the slant angle of the magnetic field causes a slight increase in velocity. In an effort to improve heat transfer, Tusi et al. ^29^ examined the natural motion of a water fluid containing nanocopper particles in a square cavity that was partially filled with porous media. They utilized the Darcy-Brinkman-Forchheimer relationship for fluid flow via porous media and the two-phase mixture model for simulating nanofluid flow. Additionally, they looked into how fluid flow and heat transfer were affected by the concentration of nanoparticles, Rayleigh and Darcy numbers, and the thickness ratio of the porous layer. Geridonmez et al. ^30–32^ investigated the mathematical analysis of natural convection flow in a square cavity that is exposed to a constant magnetic field. The RBF method for spatial derivatives and the backward Euler method for time derivatives are used to discretize the governing dimensionless equations. The findings demonstrate that the Lorentz force significantly reduces fluid flow and heat transfer in the affected area. In summary, this study provides significant findings regarding the aforementioned aspects. Convective heat transfer is decreased, and fluid flows more slowly as the influence area grows because of the increase in Lorentz force. As they concluded, the applied magnetic field is capable of controlling fluid flow and heat transfer. In an effort to enhance heat transfer, Several investigations were carried out to examine a cavity under various circumstances when a magnetic field was present ^33–47^. Alsabery et al. ^48–50^ did several works in the field of heat transfer inside the porous cavity.

This paper explores the natural convection in a square, wavy, porous cavity with a partial magnetic field. Our objective is to analyze the fluid flow patterns and heat transfer behavior within the cavity through numerical simulations and mathematical modeling. This study supports existing knowledge by providing information on the influence of external factors, such as magnetic fields and porous media, on natural convection phenomena.

## Problem formulation

**Figure 1:**
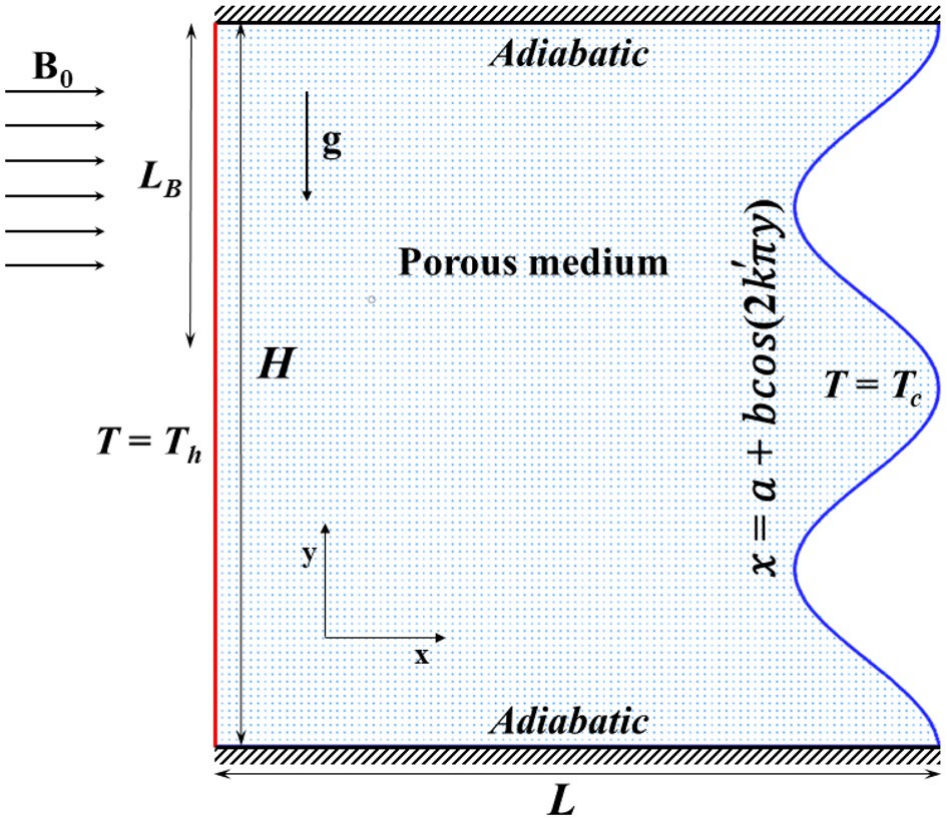
Definition of problem geometry.

A cavity with porous material and a partially imposed magnetic field is being considered for a laminar, incompressible natural convection flow. The configuration of the problem can be seen in Fig. 1. The cavity’s top and bottom walls are thermally insulated, while the left wall is the heat source (T = T_h_), and the right wavy wall is the cold boundary (T = T_c_). The cavity’s length (L) and height (H) are in unity. The values of the parameters related to the right wall are: a = 0.9, b = 0.1, and $k{\prime}$ is the wave number. The porous material inside the cavity is uniform and has isotropic properties. The fluid and porous material reach a local thermal equilibrium. The current analysis does not account for the effects of radiation effects, viscous dissipation, Joule heating, or the induced magnetic field.

Darcy’s law alone may not be sufficient in various scenarios, such as when dealing with more porous materials, high velocities, or high Reynolds number effects. In 1901, Forchheimer introduced the concept of a quadratic drag term (also known as the inertial or Forchheimer term) to Darcy’s law. Later, Brinkmann further expanded on the model to incorporate larger porosity and account for viscous effects. It’s worth mentioning that this study excludes certain factors, such as induced magnetic fields and Joule heating.

Brinkman-Forchheimer-extended Darcy’s model is utilized in this study to analyze the porous material within the cavity. The problem’s configuration involves a laminar, incompressible natural convection flow with a partially applied magnetic field. This study’s governing equations are the continuity, momentum, and energy expressed in the u-v-p-T form. These nonlinear equations capture the complex dynamics of the system and provide insights into the fluid flow, pressure distribution, and temperature distribution within the porous material-filled cavity, as follows ^31^:

1 $$\nabla .\mathbf{u}=0,$$

2 $$\frac{{\mu }_{e}}{{\rho }_{f}{\epsilon }_{p}}{\nabla }^{2}u=\frac{1}{{\epsilon }_{p}}\frac{\partial u}{\partial t}+\frac{1}{{{\epsilon }_{p}}^{2}}\left(u\frac{\partial u}{\partial x}+v\frac{\partial u}{\partial y}\right)+\frac{1}{{\rho }_{f}}\frac{\partial p}{\partial x}+\frac{\mu }{{\rho }_{f}\mathsf{K}}u+\frac{{c}_{F}}{\sqrt{\mathsf{K}}}\left|\mathbf{u}\right|u,$$

3 $$\frac{{\mu }_{e}}{{\rho }_{f}{\epsilon }_{p}}{\nabla }^{2}v=\frac{1}{{\epsilon }_{p}}\frac{\partial v}{\partial t}+\frac{1}{{{\epsilon }_{p}}^{2}}\left(u\frac{\partial v}{\partial x}+v\frac{\partial v}{\partial y}\right)+\frac{1}{{\rho }_{f}}\frac{\partial p}{\partial y}+\frac{\mu }{{\rho }_{f}k}v+\frac{{c}_{F}}{\sqrt{\mathsf{K}}}\left|\mathbf{u}\right|v-g\beta \left(T-{T}_{c}\right)+{\updelta }_{B}\frac{\upsigma {{B}_{0}}^{2}}{{\rho }_{f}}v,$$

4 $${{\alpha }}_{e}{\nabla }^{2}T={\upsigma }_{h}\frac{\partial T}{\partial t}+\mathbf{u}.\nabla T.$$

In which $\mu$ corresponds to the dynamic viscosity of the fluid, the effective dynamic viscosity is ${\mu }_{e}$, the fluid’s density is ${\rho }_{f}$, the norm of the velocity vector $\sqrt{({u}^{2}+{v}^{2})}$ is $\left|\mathbf{u}\right|$, $g$ is the gravitational acceleration, the thermal expansion coefficient is $\beta$, the pressure is defined by $p$, the porosity of a porous medium is measured by ${\epsilon }_{p}$, the magnitude of the applied magnetic field is referred to as ${B}_{0}$, the fluid’s electrical conductivity is defined as σ, the effective thermal diffusivity is ${{\alpha }}_{e}=\frac{{k}_{e}}{{({\rho }_{f}{c}_{p})}_{f}}$, the effective thermal conductivity is ${k}_{e}={\epsilon }_{p}{k}_{f}+(1-{\epsilon }_{p}){k}_{s}$, the specific heat at constant pressure is ${c}_{p}$, the coefficient of form is ${c}_{F}=\frac{1.75(1-{\epsilon }_{p})}{{d}_{p}{{\epsilon }_{p}}^{3}}$, $K=\frac{{{d}_{p}}^{2}{{\epsilon }_{p}}^{3}}{150{(1-{\epsilon }_{p})}^{2}}$ refers to the permeability of the porous medium, ${d}_{p}$ is the size of a solid particle in a porous medium, and the heat capacity ratio ${\upsigma }_{h}=\frac{{\epsilon }_{p}{({\rho }_{f}{c}_{p})}_{f} +(1-{\epsilon }_{p}){({\rho }_{f}{c}_{p})}_{s}}{{({\rho }_{f}{c}_{p})}_{f}}$ , is considered one in this model. Additionally, the fluid $(f)$ and solid $(s)$ are assumed to have the same thermal conductivity and thermal diffusivity. That means, ${k}_{e}={k}_{f}={k}_{s}$ and ${\alpha }_{e}={\alpha }_{f}=\alpha$. In addition, ${\mu }_{e}=\mu$ is assumed. Air fluid was used for the present work. The physical properties of air are given in Table 1.

**Table 1:** Physical properties of air at 20^◦^c.

Parameter	${{\varvec{\rho}}}_{{\varvec{f}}}$	${{\varvec{\mu}}}_{{\varvec{f}}}$	${{\varvec{c}}}_{{\varvec{p}}}$	${\varvec{k}}$	${\varvec{\sigma}}$
Physical value	1.2047 kg/m^3^	1.820 × 10^−5^ kg/m.s	1000 J/kg.K	0.02559 W/m.K	5 × 10^−15^

The boundary conditions are outlined below:

5a $$u=v=0, \frac{\partial T}{\partial y}=0 at y=\text{0,1} , 0<x<1,$$

5b $$u=v=0, T={T}_{h} at x=0 , 0<y<1,$$

5c $$u=v=0, T={T}_{c} at x=a+bcos(2k\pi y) , 0<y<1.$$

The dimensionless quantities are defined below:

6a $${x}{\prime}=\frac{x}{L} , {y}{\prime}=\frac{y}{L} , {u}{\prime}=\frac{uL}{\alpha } , {v}{\prime}=\frac{vL}{\alpha } ,$$

6b $${p}{\prime}=\frac{p{L}^{2}}{{\rho }_{f}{\alpha }^{2}} ,{ t}{\prime}=\frac{t\alpha }{{L}^{2}} , \theta =\frac{T-{T}_{c}}{{T}_{h}-{T}_{c}}.$$

With the help of non-dimensional quantities, they are replaced in the original Eqs. (1)-(4), and assuming the elimination of prime symbols, the following dimensionless equations are obtained.

7 $$\frac{\partial u}{\partial x}+\frac{\partial v}{\partial y}=0,$$

8 $$\frac{Pr}{{\epsilon }_{p}}{\nabla }^{2}u=\frac{1}{{\epsilon }_{p}}\frac{\partial u}{\partial t}+\frac{1}{{{\epsilon }_{p}}^{2}}\left(u\frac{\partial u}{\partial x}+v\frac{\partial u}{\partial y}\right)+\frac{\partial p}{\partial x}+\frac{Pr}{Da}u+{c}_{g}\frac{\left|\mathbf{u}\right|}{\sqrt{Da}}u,$$

9 $$\frac{Pr}{{\epsilon }_{p}}{\nabla }^{2}v=\frac{1}{{\epsilon }_{p}}\frac{\partial v}{\partial t}+\frac{1}{{{\epsilon }_{p}}^{2}}\left(u\frac{\partial v}{\partial x}+v\frac{\partial v}{\partial y}\right)+\frac{\partial p}{\partial y}+\frac{Pr}{Da}v+{c}_{g}\frac{\left|\mathbf{u}\right|}{\sqrt{Da}}v-RaPr\theta +{\updelta }_{B}{Ha}^{2}Prv,$$

10 $${\nabla }^{2}\theta =\frac{\partial \theta }{\partial t}+u\frac{\partial \theta }{\partial x}+v\frac{\partial \theta }{\partial y}.$$

where, ${c}_{g}=\frac{ 1.75}{\sqrt{150}{{\epsilon }_{p}}^{3/2}}$. The dimensionless parameters Prandtl, Darcy, Rayleigh, and Hartmann numbers are as follows:

11 $$ \Pr  = {\raise0.7ex\hbox{$\mu $} \!\mathord{\left/ {\vphantom {\mu  {\alpha \rho _{f} }}}\right.\kern-\nulldelimiterspace} \!\lower0.7ex\hbox{${\alpha \rho _{f} }$}},\,\,\,\,Da = {\raise0.7ex\hbox{$K$} \!\mathord{\left/ {\vphantom {K {L^{2} }}}\right.\kern-\nulldelimiterspace} \!\lower0.7ex\hbox{${L^{2} }$}},\,\,\,\,Ra = {\raise0.7ex\hbox{${g\beta \Delta T\rho _{f} L^{3} }$} \!\mathord{\left/ {\vphantom {{g\beta \Delta T\rho _{f} L^{3} } {\mu \alpha }}}\right.\kern-\nulldelimiterspace} \!\lower0.7ex\hbox{${\mu \alpha }$}},\,\,\,\,Ha = B_{0} L\sqrt {{\raise0.7ex\hbox{$\sigma $} \!\mathord{\left/ {\vphantom {\sigma  \mu }}\right.\kern-\nulldelimiterspace} \!\lower0.7ex\hbox{$\mu $}}} . $$

where $\Delta T={T}_{h}-{T}_{c}$. By setting the stream function $\psi$ as $u=\partial \psi /\partial y, v=-\partial \psi /\partial x$, it eliminates the continuity equation due to satisfying the continuity condition, and the pressure term is removed by using the vorticity definition $\omega =\nabla \times \mathbf{u}$ in the momentum equations. Non-dimension equations are deduced from the effects of stream and vorticity functions:

12 $${\nabla }^{2}\psi =\omega ,$$

13 $${\nabla }^{2}\omega =\frac{1}{Pr}\frac{\partial \omega }{\partial t}+\frac{1}{Pr{\epsilon }_{p}}\left(u\frac{\partial \omega }{\partial x}+v\frac{\partial \omega }{\partial y}\right)+\frac{{\epsilon }_{p}}{Da}\omega +\frac{{c}_{g}{\epsilon }_{p}}{Pr\sqrt{Da}}\left[v\frac{\partial \left|\mathbf{u}\right|}{\partial x}-u\frac{\partial \left|\mathbf{u}\right|}{\partial y}+\left|\mathbf{u}\right|\omega \right]-{\epsilon }_{p}Ra\frac{\partial \theta }{\partial x}+{\epsilon }_{p}{\updelta }_{B}{Ha}^{2}\frac{\partial v}{\partial x},$$

14 $${\nabla }^{2}\theta =\frac{\partial \theta }{\partial t}+u\frac{\partial \theta }{\partial x}+v\frac{\partial \theta }{\partial y}.$$

In the case of the incoming magnetic field, ${\delta }_{B}$ , is defined as:

15 $${\delta }_{B}=\left\{\begin{array}{c}0, 0\le y<1-{L}_{B}\\ 1, 1-{L}_{B}\le y<1.\end{array}\right.$$

The reduced boundary conditions are as follows:

16a $$\psi =0, \frac{\partial \theta }{\partial y}=0 at y=\text{0,1} , 0<x<1,$$

16b $$\psi =0, \theta =1 at x=0 , 0<y<1,$$

16c $$\psi =0, \theta =0 at x=a+bcos(2k\pi y) , 0<y<1,$$

The average Nusselt number for the warm wall is determined as follows:

17 $$\overline{Nu }=\frac{1}{H}{\int }_{0}^{H}-\frac{\partial \theta }{\partial x}dy.$$

## Method of solution and numerical results

**Figure 2:**
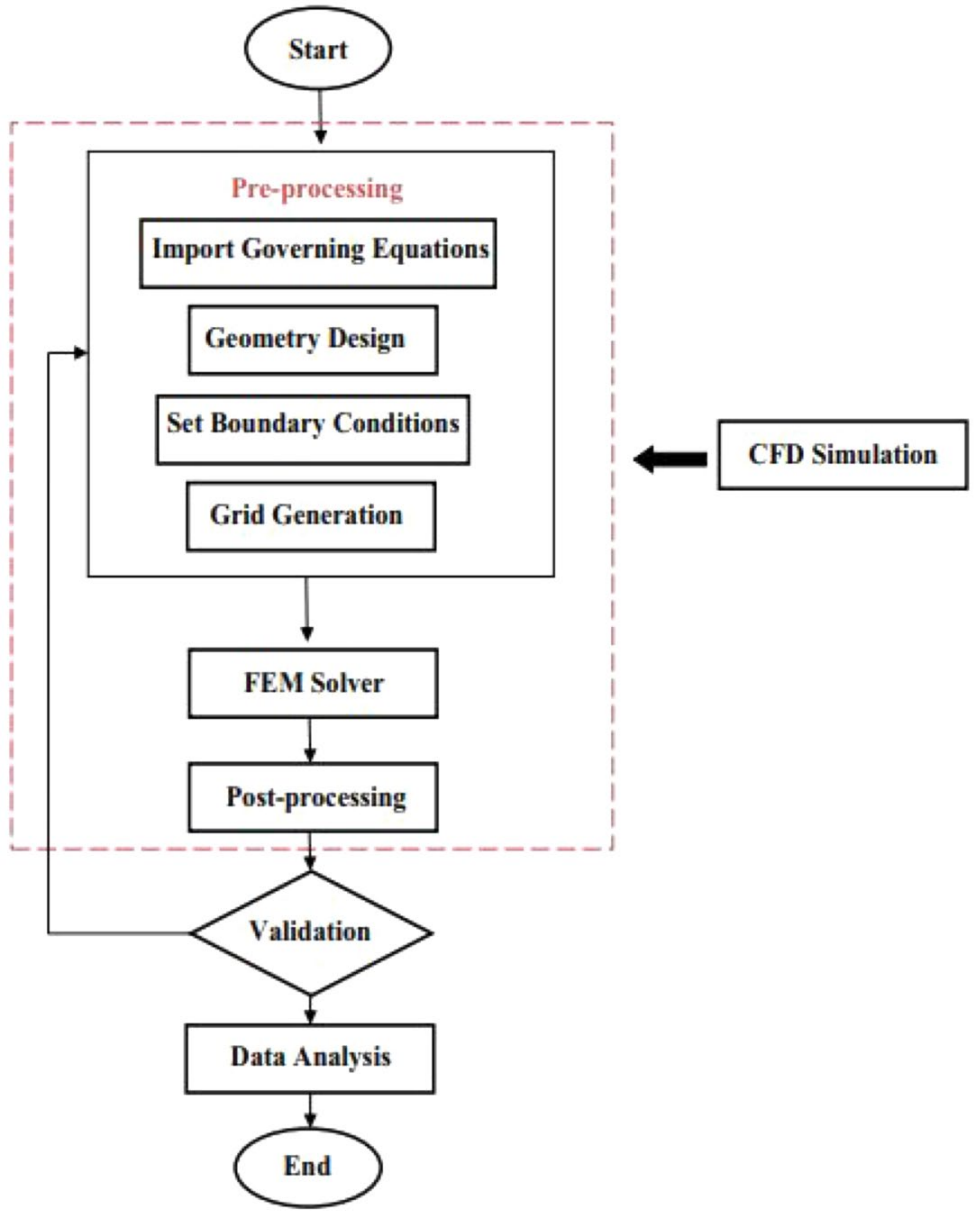
The flowchart of the numerical method.

**Figure 3:**
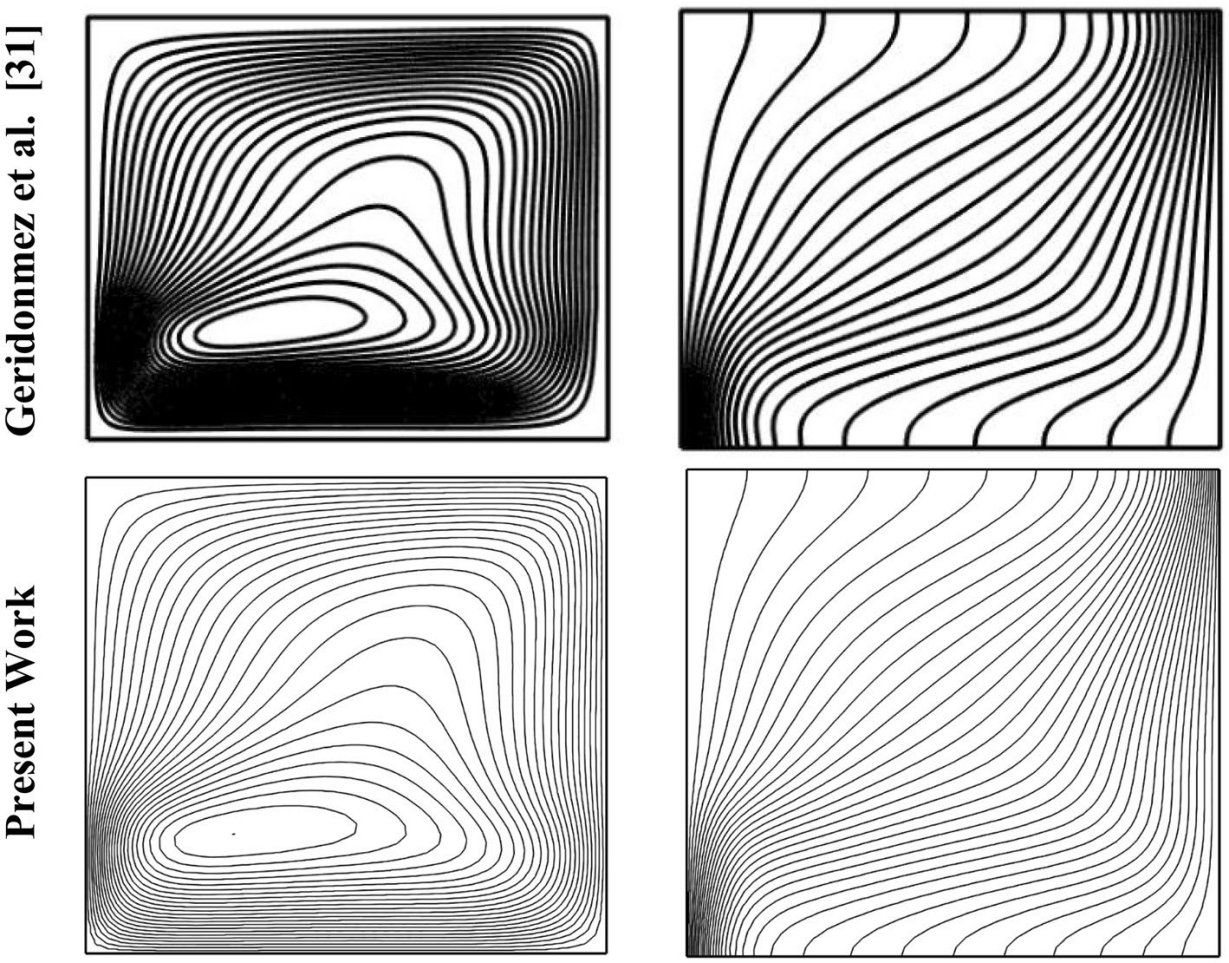
Comparison of streamlines (on the left) and isotherm lines (on the right) at Ra = 10^5^, Da = 0.01, ϵ_p_ = 0.9, Ha = 50, L_B_ = 0.7.

The Eqs. (12)-(14) have been solved using the Finite Element Method with boundary conditions (16). The reliability of this method is a result of its high strength and flexibility. Once the initial meshing is done, the solution continues continuously. To achieve acceptable accuracy, it may also alter the mesh structure. This process continues until the convergence condition is met. The convergence condition for the present work is to reach 10^–5^ accuracy. The Finite Element Method (FEM) operates on a fundamental principle: breaking down a complex problem domain into discrete sub-regions that are called finite elements. Each of these elements possesses a distinct geometry and is characterized mathematically through a set of equations that describe the behavior of the system within that particular section. This approach simplifies the solution of complex governing equations, which would be arduous or nearly impossible to solve manually. Shape functions are used in FEM to interpolate element-level solutions based on nodal values. In this study, FEM serves as a numerical tool for solving the governing equations related to fluid flow and heat transfer due to natural convection of a square wavy cavity with a magnetic field and porous media. To improve understanding of the numerical solution technique, Fig. 2 displays the flowchart of the numerical method. Through FEM, a more precise and detailed solution for the natural convection of a square wavy cavity with a magnetic field and porous media can be obtained. Figure 3 shows a good match between the streamlines and isotherm lines compared to the work of Geridonmez et al. ^31^.

**Figure 4:**
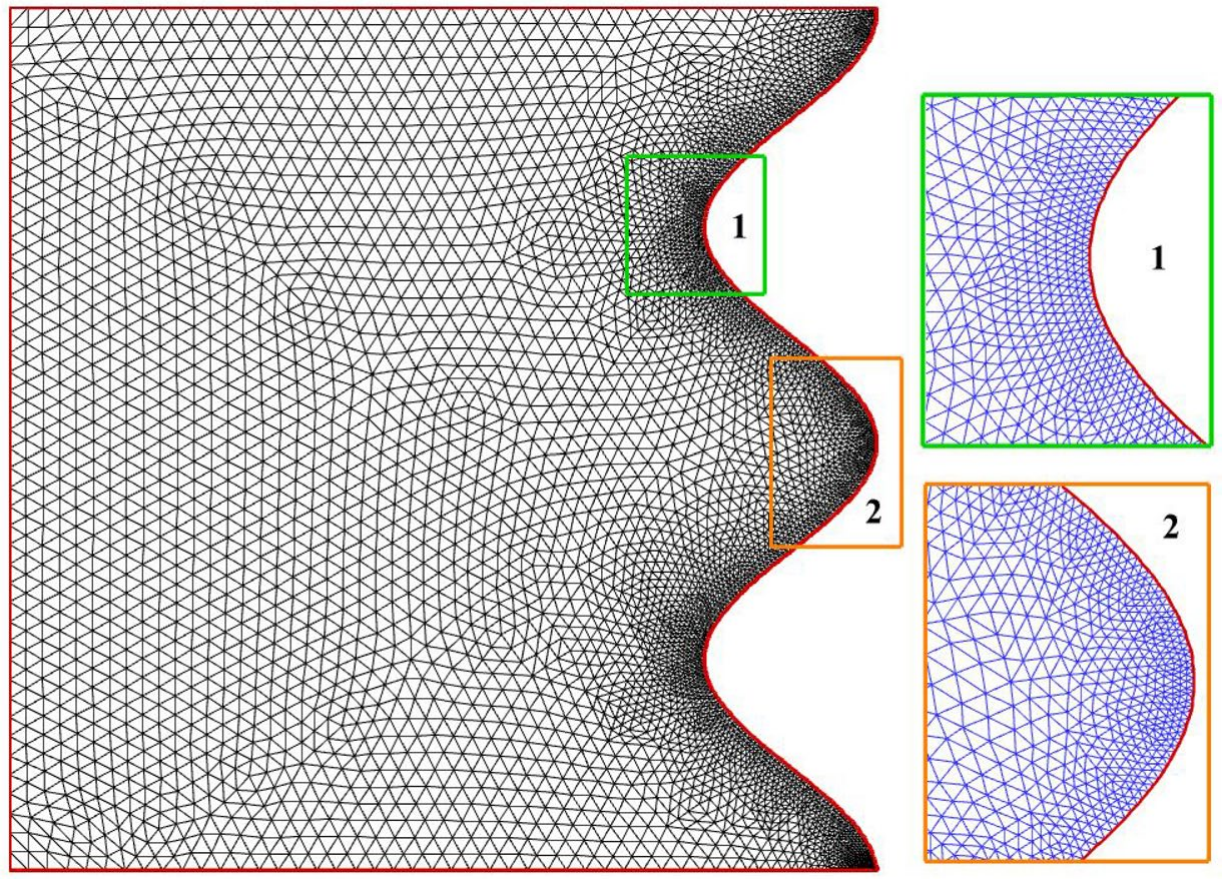
Grid of geometry.

In order to obtain a mesh-independent solution, mesh independence is investigated. The grid-independent solution was examined by providing a solution for natural convection in a square cavity with one side having a cosine wave with a magnetic field applied to a portion of the square side. Five of the grids have been tested. As can be seen in Table 2, with the number of cells 3006, the solution becomes independent and reaches an accuracy of 10^–5^. The optimal mesh is shown in Fig. 4.

**Table 2:** Mesh independent review with Ra = 10^5^, Da = 0.01, ϵ_p_ = 0.9, Ha = 50, L_B_ = 0.5, $k{\prime}$ = 2.

Cells	1139	2187	3006	4592	6640
$\overline{\text{Nu} }$	4.24882	4.24652	4.24522	4.24514	4.24502
${\left|\Psi \right|}_{\text{Max}}$	7.01255	7.00847	7.00535	7.00522	7.00521

## Results and discussions

**Figure 5:**
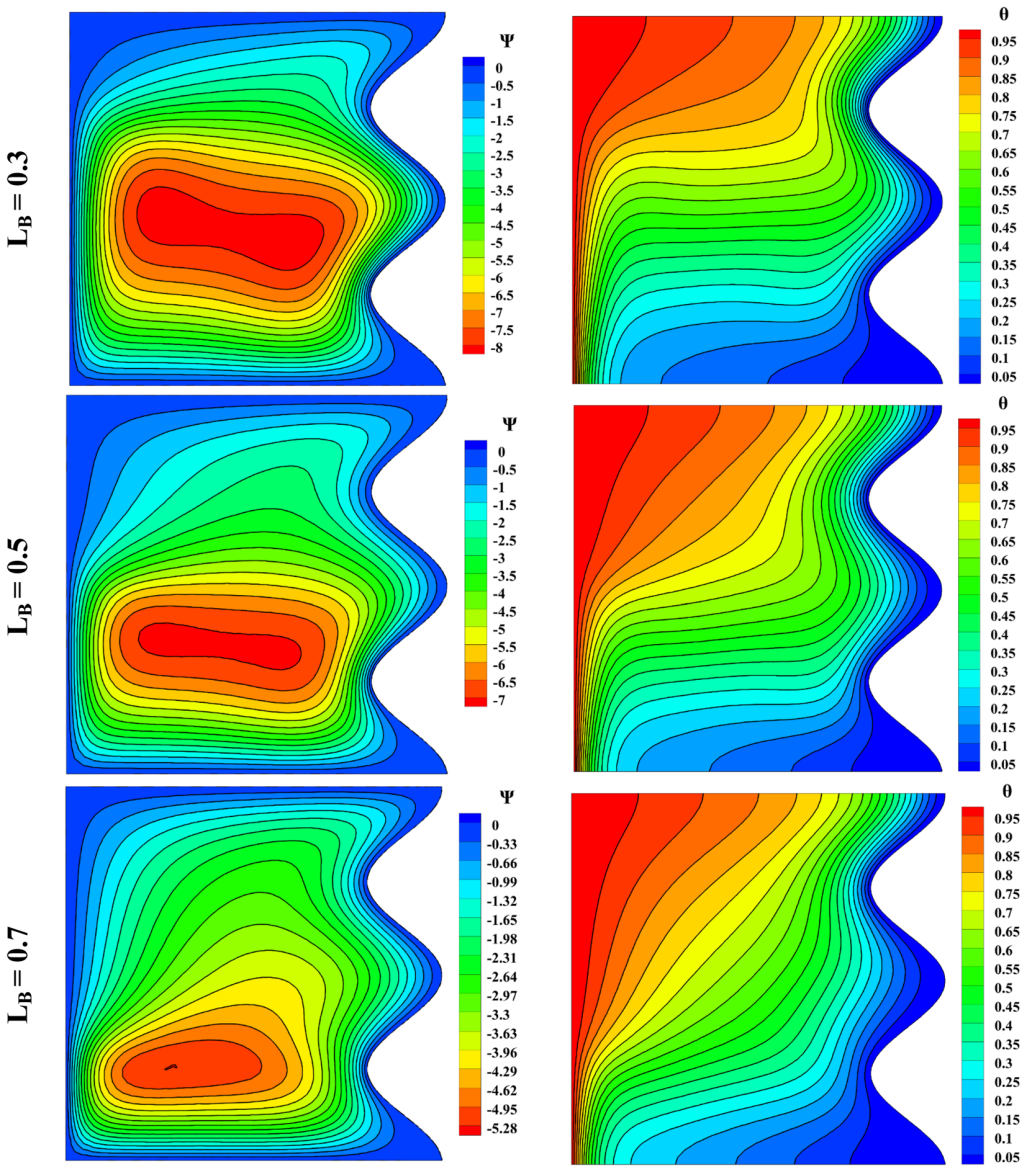
Impact of magnetic field length on streamlines and isotherm lines with Ra = 10^5^, Da = 0.01, ϵ_p_ = 0.9, Ha = 50, $k{\prime}$= 2; from top to bottom Nu = 5.127, 4.245, 3.247, respectively.

Figure 5 shows the impact of the entranced magnetic field length on the left wall. As the magnetic field length increases, the main vortex is compressed towards the lower left nook of the cavity, but the upper part of the vortex is stretched towards the upper right nook of the cavity because of the increased Lorentz force area introduced by the magnetic field. The vortex’s strength decreases with the magnetic field’s length. On the other hand, the buoyancy force slows down. The temperature difference in the isotherms decreases. In addition, the isotherm lines tend to flatten, indicating the magnetic force’s inhibition of natural convection.

**Figure 6:**
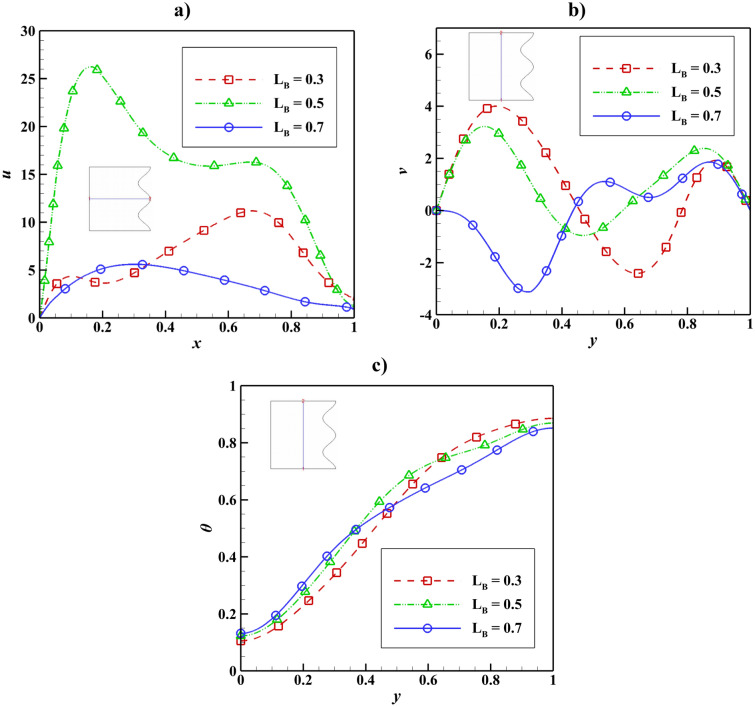
Velocity and temperature distribution based on magnetic field length change with Ra = 10^5^, Da = 0.01, ϵ_p_ = 0.9, Ha = 50, $k{\prime}$ = 2; (**a**) *u* at y = 0.5, (**b**) *v* at x = 0.5, (**c**) *θ* at x = 0.5.

The velocity and temperature distribution based on the variable magnetic field length can be seen in Fig. 6. It is concluded that by raising the magnetic field length, the velocity decreases, and the temperature increases from the bottom of the cavity to its center, but this process is the opposite for the upper half. The maximum velocity *u* belongs to L_B_ = 0.5.

**Figure 7:**
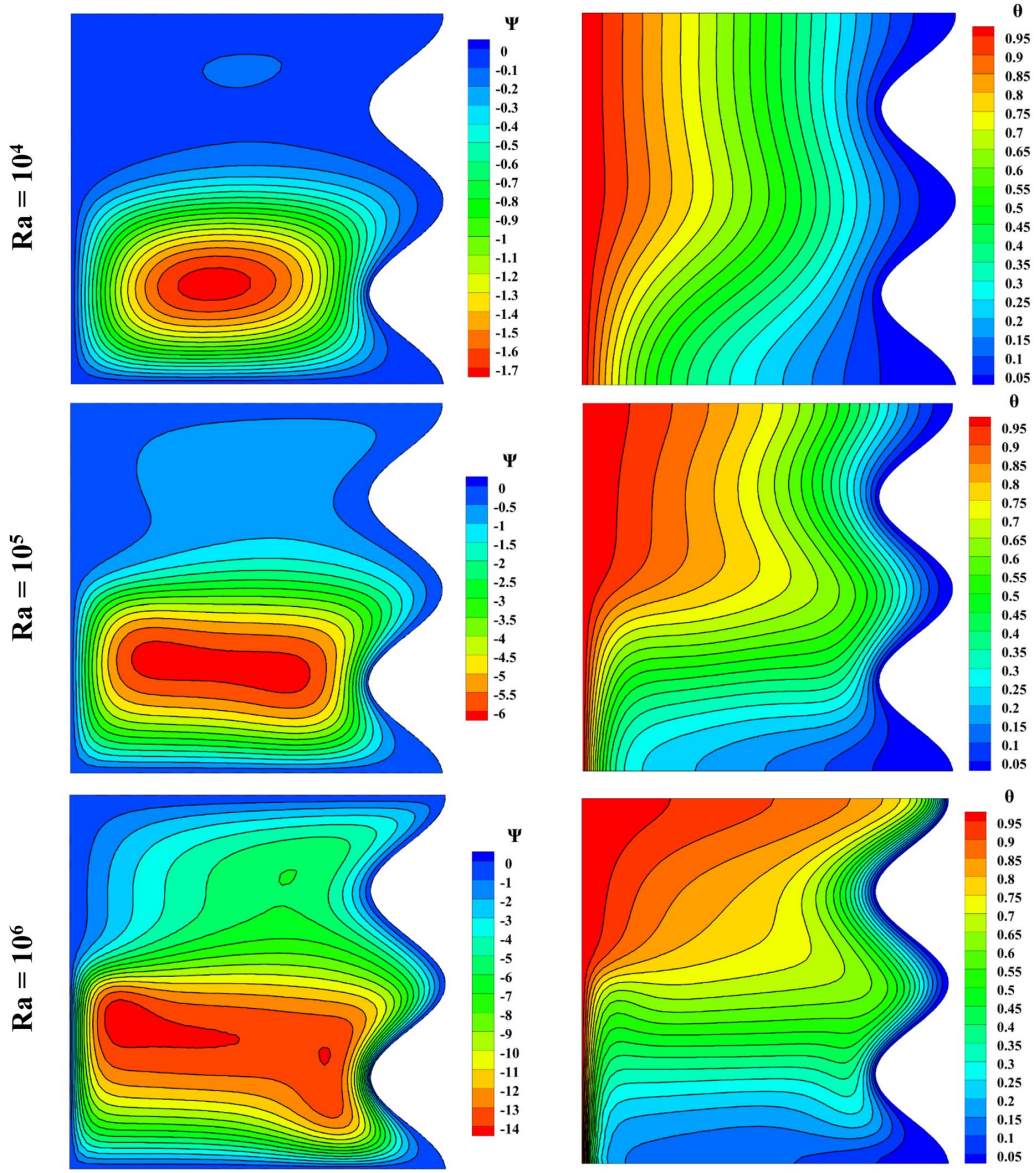
Influence of Rayleigh number on streamlines and isotherm lines with Da = 0.01, ϵ_p_ = 0.9, Ha = 100, L_B_ = 0.5, $k{\prime}$ = 2; from top to bottom Nu = 1.465, 3.6, 8.944, respectively.

Figure 7 shows the effect of the Rayleigh number on streamlines and isothermal lines. L_B_ = 0.5, as defined, means that the magnetic field affects the left wall of the cavity from the top to the center. Therefore, the streamlines show the natural convection at the bottom of the cavity, while the upper part of the cavity shows the lagging impact of the Lorentz force. Because of the dominance of Lorentz force over the buoyancy force, the second vortex is seen in the streamlines at Ra = 10^4^, while it is not observed at Ra = 10^5^ or 10^6^ due to the high buoyancy force. The isotherm lines are approximately 90 degrees to the upper wall for Ra = 10^4^, 10^5^. With the increase of Rayleigh, the isotherm lines tend to bend more from the flat state, and heat penetrates more in the upper half.

**Figure 8:**
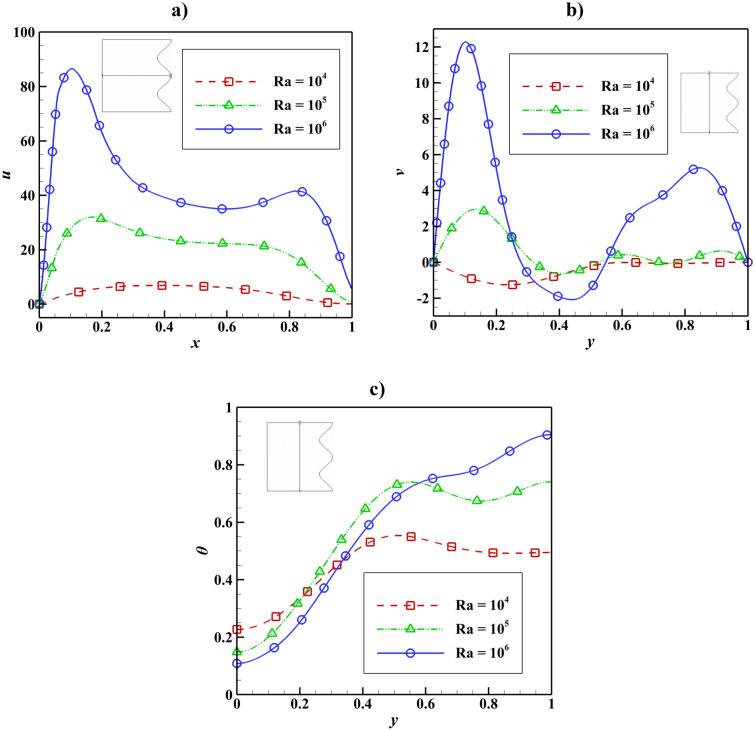
Velocity and temperature distribution based on Rayleigh number change with Da = 0.01, ϵ_p_ = 0.9, Ha = 100, L_B_ = 0.5, $k{\prime}$ = 2; (**a**) *u* at y = 0.5, (**b**) *v* at x = 0.5, (**c**) *θ* at x = 0.5.

The velocity and temperature distribution based on the Rayleigh number change can be seen in Fig. 8. Raising the Rayleigh number lowered the temperature in the cavity’s lower region while increasing the maximum velocity, but this trend changes for the upper part of the cavity, and the temperature increases.

**Figure 9:**
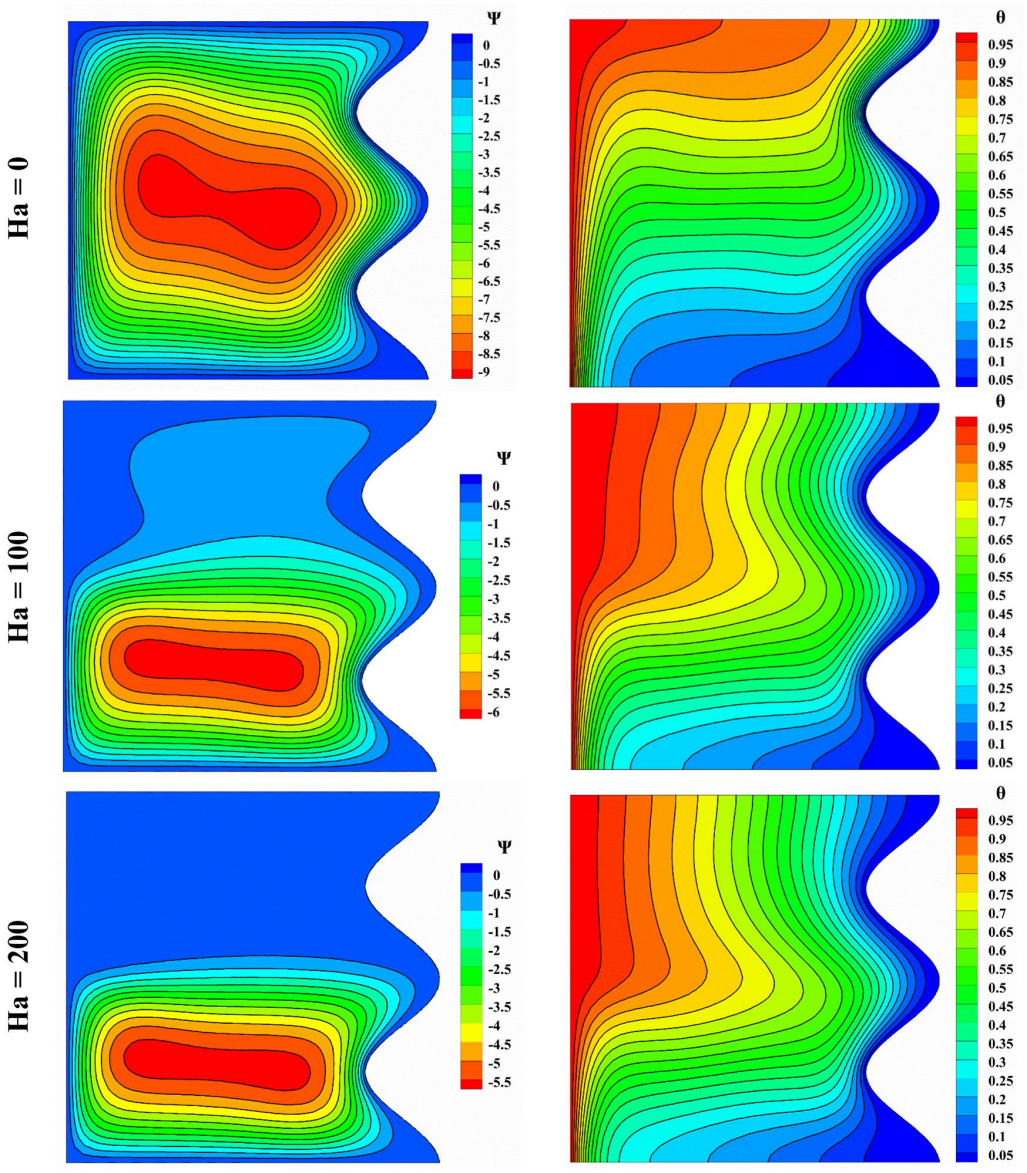
Impact of Hartmann number on streamlines and isotherm lines with Da = 0.01, ϵ_p_ = 0.9, Ra = 10^5^, L_B_ = 0.5, $k{\prime}$ = 2; from top to bottom Nu = 5.973, 3.6, 3.342, respectively.

Figure 9 shows the effect of the Hartmann number on streamlines and isothermal lines. In case there’s no magnetic field due to the non-existence of Lorentz force delay, the streamlines show natural convection in all parts of the cavity. With the augmentation of the Hartmann number, the vortex tends to the lower side of the cavity. Also, the increase in Hartmann reduces the strength of the vortex.

The isotherm lines are almost perpendicular to the top wall for Ra = 10^5^, 10^6^. As the Rayleigh increases, the isotherm lines tend to bend less from the flat state, and heat penetrates less in the upper half, indicating that the magnetic field was controlling the heat transfer and fluid flow.

**Figure 10:**
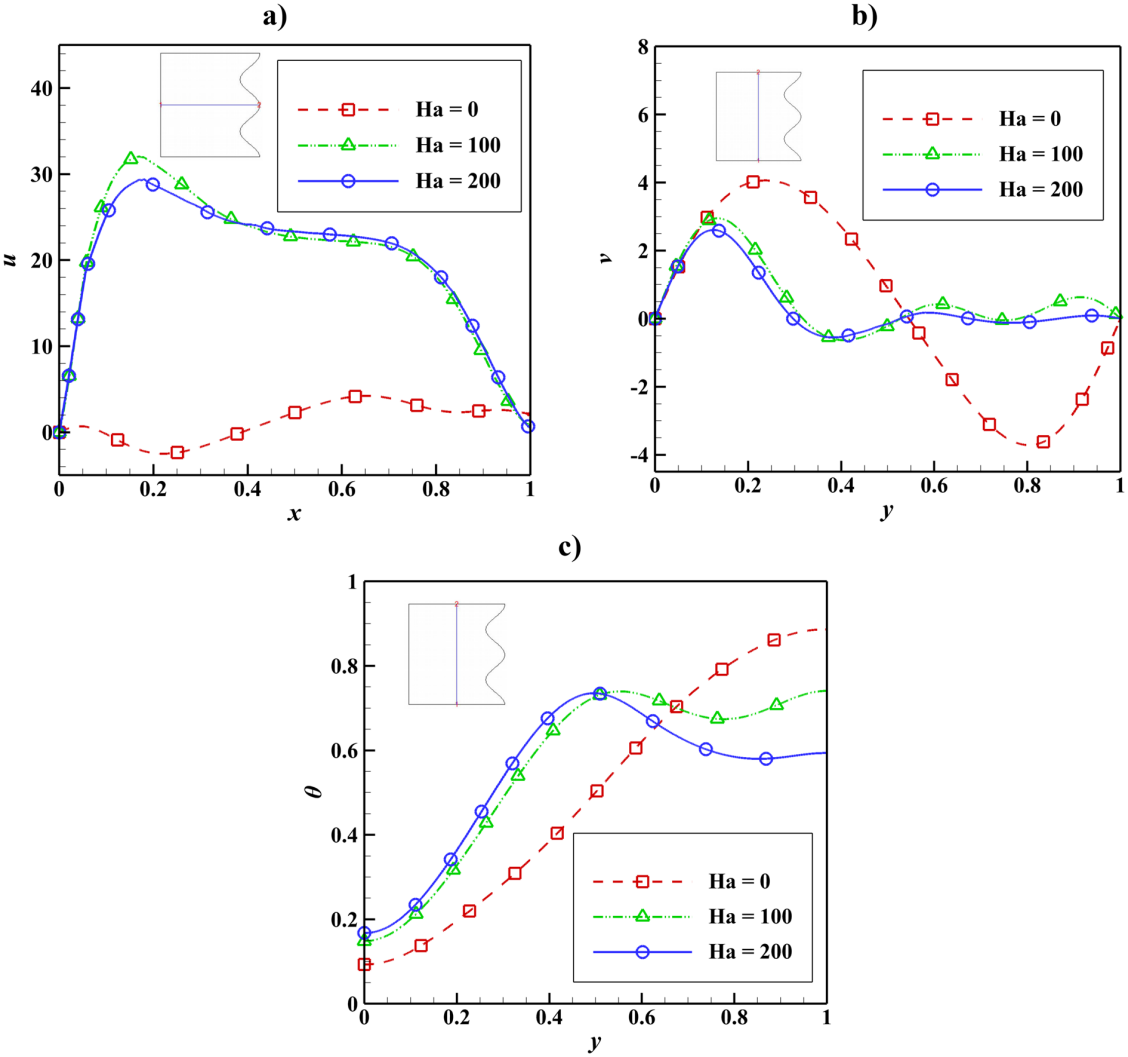
Velocity and temperature distribution based on Hartmann number change with Da = 0.01, ϵ_p_ = 0.9, Ra = 10^5^, L_B_ = 0.5, $k{\prime}$ = 2; (**a**) *u* at y = 0.5, (**b**) *v* at x = 0.5, (**c**) *θ* at x = 0.5.

The velocity and temperature distribution due to the Hartmann number change is shown in Fig. 10. In the absence of a magnetic field, *u* has the lowest value, and with the augmentation of the Hartmann number, *u* and *v* decrease. With increasing the Hartmann number, the temperature trend for the cavity’s lower half increases, but this trend is reversed for the upper half, and the temperature decreases.

**Figure 11:**
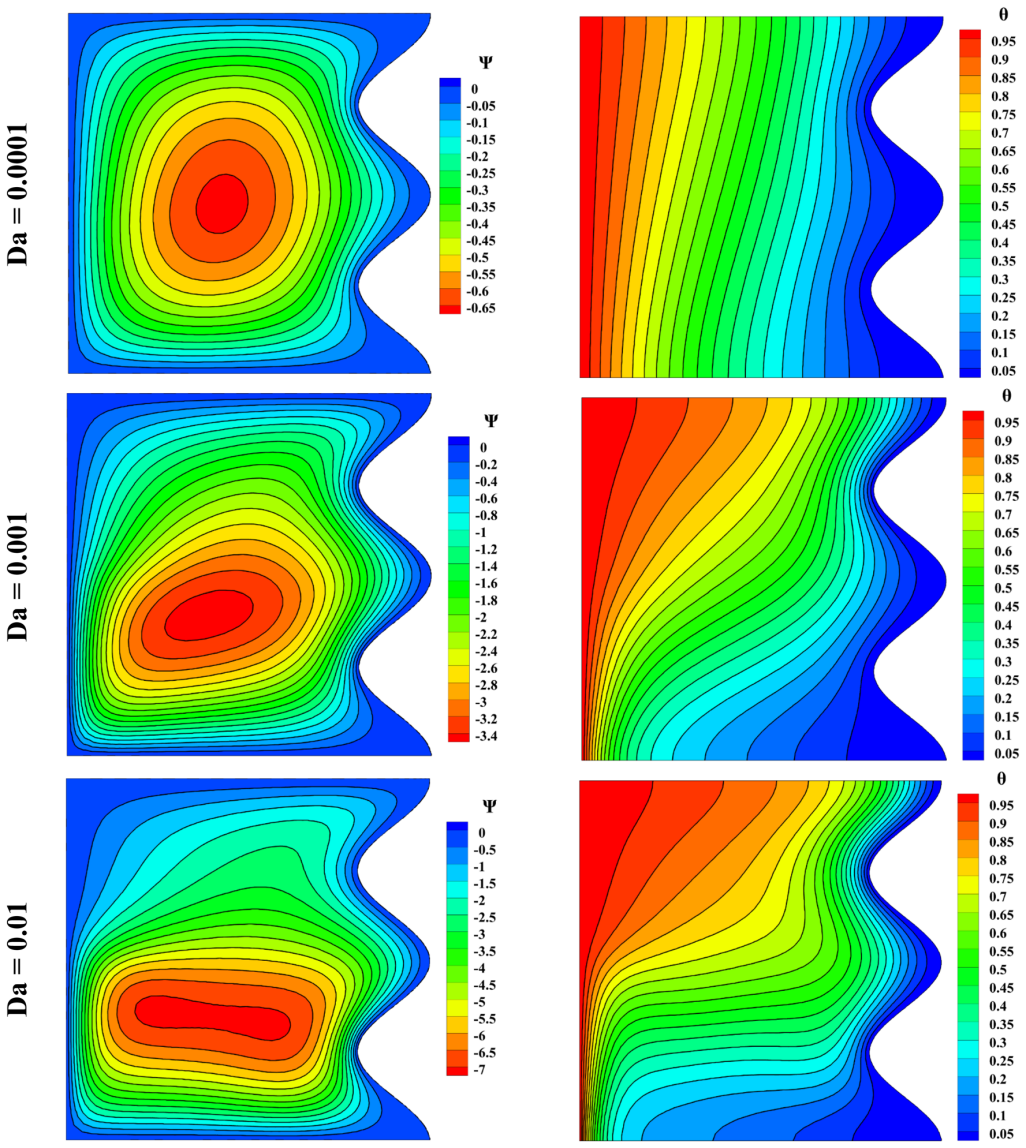
Impact of Darcy number on streamlines and isotherm lines with Ra = 10^5^, ϵ_p_ = 0.9, Ha = 50, L_B_ = 0.5, $k{\prime}$ = 2; from top to bottom Nu = 1.234, 2.404, 4.245, respectively.

The influence of Darcy number on streamlines and isotherm lines was examined, and it is shown as a contour in Fig. 11. At the Darcy number close to zero, the vorticity tends to the center, and the isothermal lines are almost parallel to each other because the Lorentz force loses its effect at a low Darcy number. With the increase of Darcy, the penetration of heat increases, and as a result, the fluid moves at a higher speed, and the vortex is transferred downwards.

**Figure 12:**
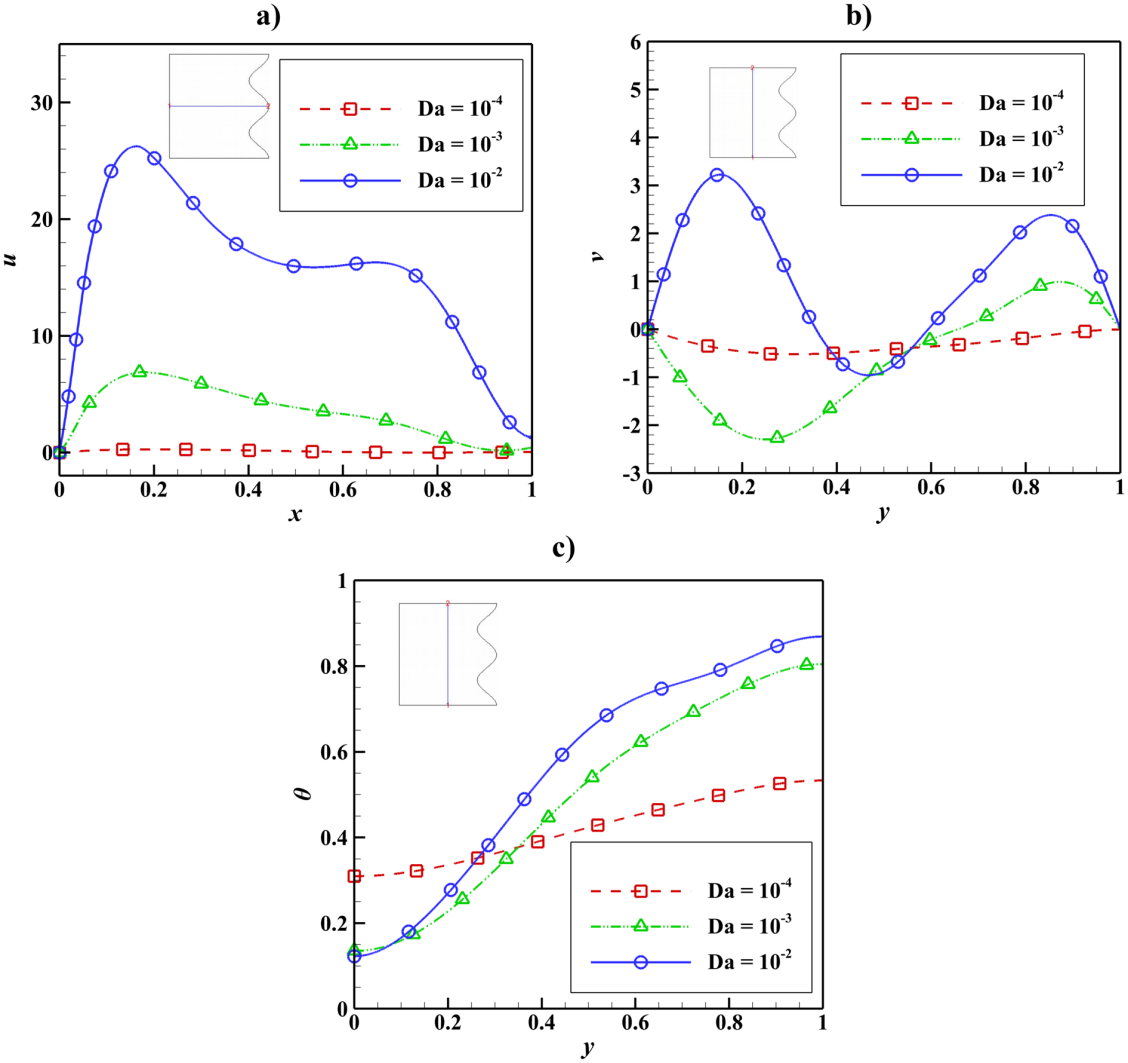
Velocity and temperature distribution based on Darcy number change with Ra = 10^5^, Ha = 50, ϵ_p_ = 0.9, L_B_ = 0.5, $k{\prime}$= 2; (**a**) *u* at y = 0.5, (**b**) *v* at x = 0.5, (**c**) *θ* at x = 0.5.

The velocity and temperature distribution due to the Darcy number change can be seen in Fig. 12. With increasing the Darcy number, the magnitude of u and v increase, and it can be said that the temperature also has an increasing trend, and in Darcy 10^–4^, the temperature changes are insignificant.

**Figure 13:**
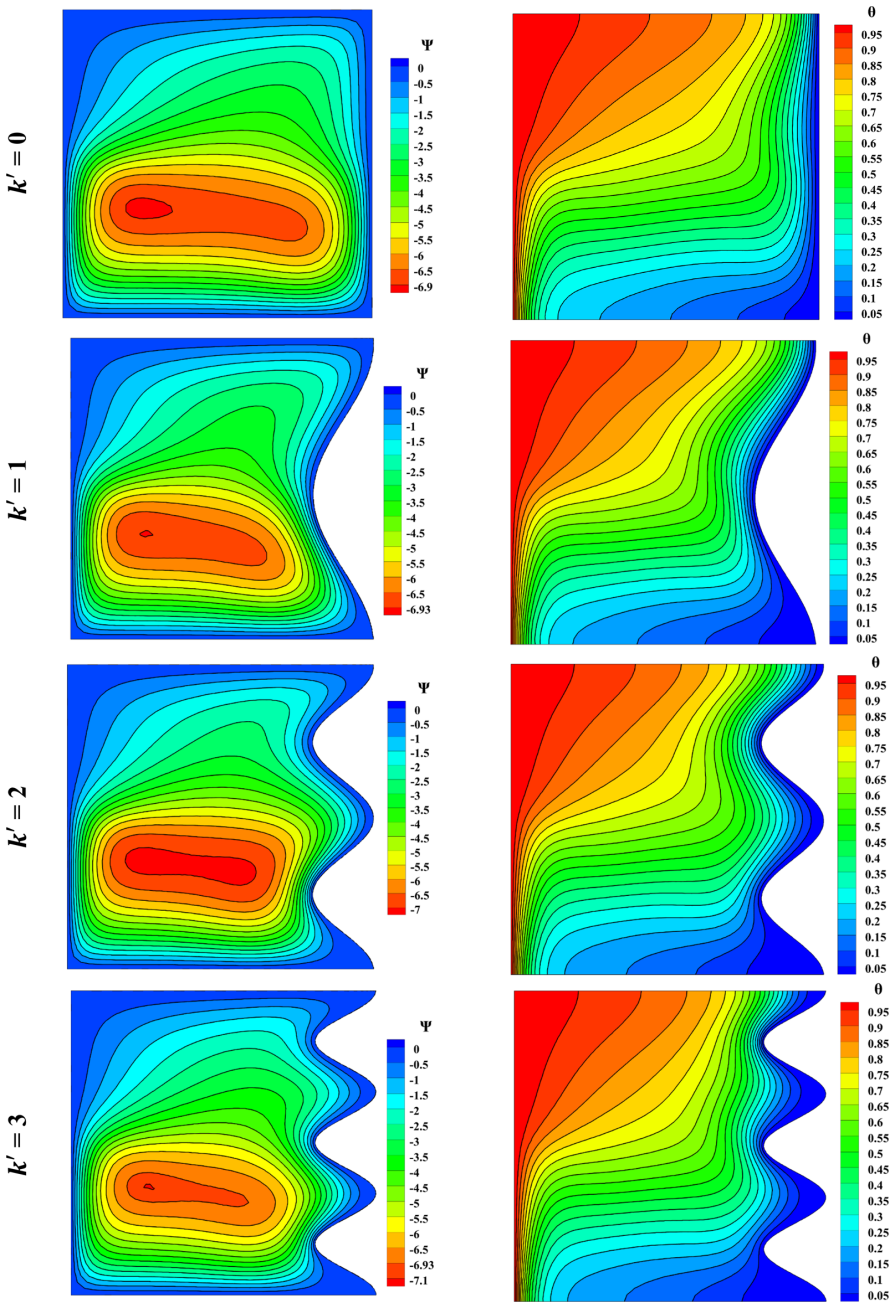
Impact of wave number on streamlines and isotherm lines with Ra = 10^5^, Da = 0.01, ϵ_p_ = 0.9, Ha = 50, L_B_ = 0.5; from top to bottom Nu = 3.978, 4.093, 4.245, 4.243, respectively.

**Figure 14:**
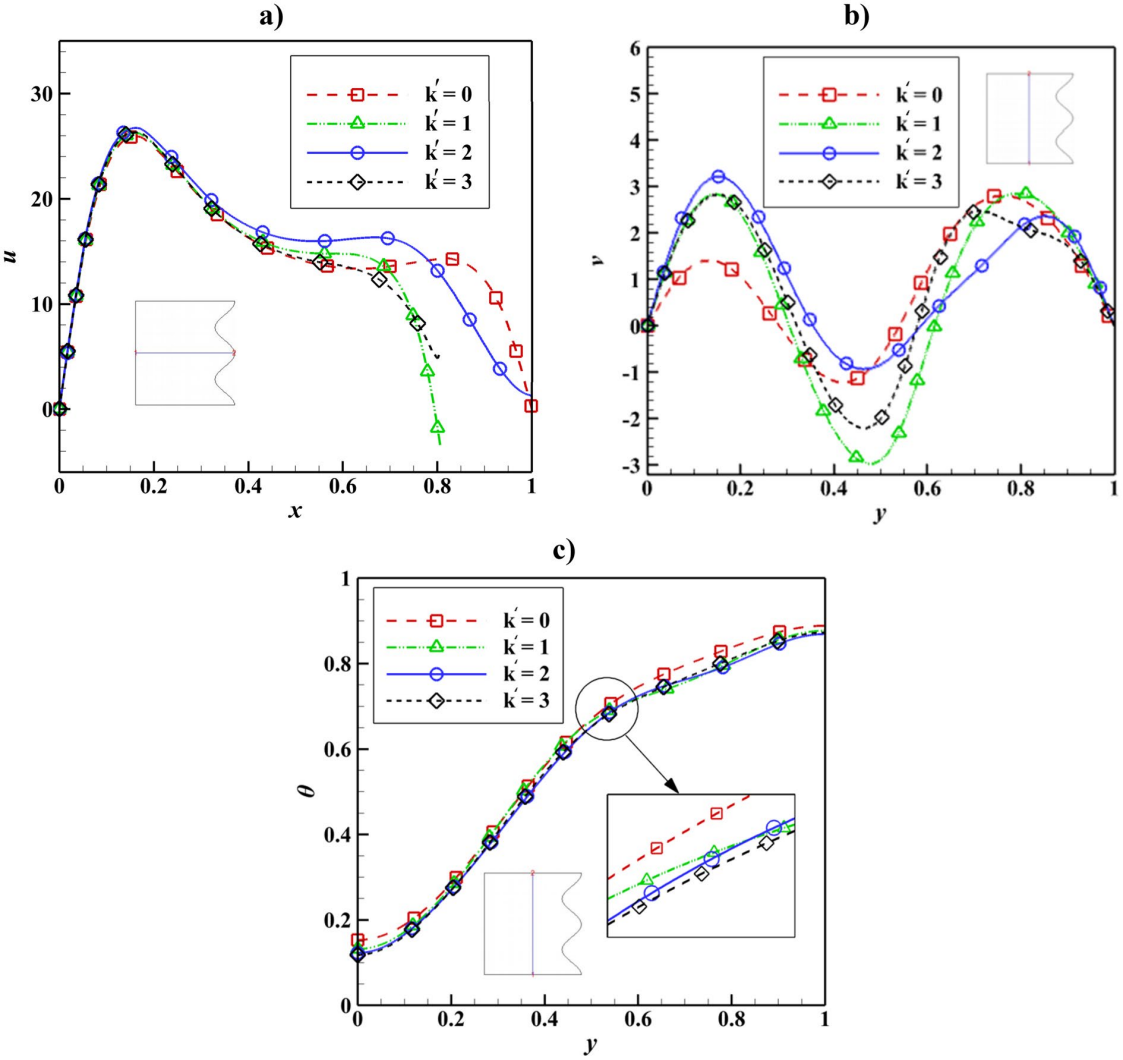
Velocity and temperature distribution based on Wave number change with Ra = 10^5^, Ha = 50, Da = 0.01, ϵ_p_ = 0.9, L_B_ = 0.5; a) *u* at y = 0.5, b) *v* at x = 0.5, c) *θ* at x = 0.5.

The impact of wave number on streamlines and isotherm lines was studied, and the outcomes are shown in Fig. 13. As the wave number increases, the eddy tends to rise less, and the fluid moves with a higher partial velocity. The number of waves has no noticeable effect on isothermal lines. Nonetheless, there is a certain increase in the Nusselt number as the wave number rises. The behavior of velocity in horizontal and vertical, as well as temperature distribution in the vertical case, is shown. See Fig. 14 for better understanding.

**Figure 15:**
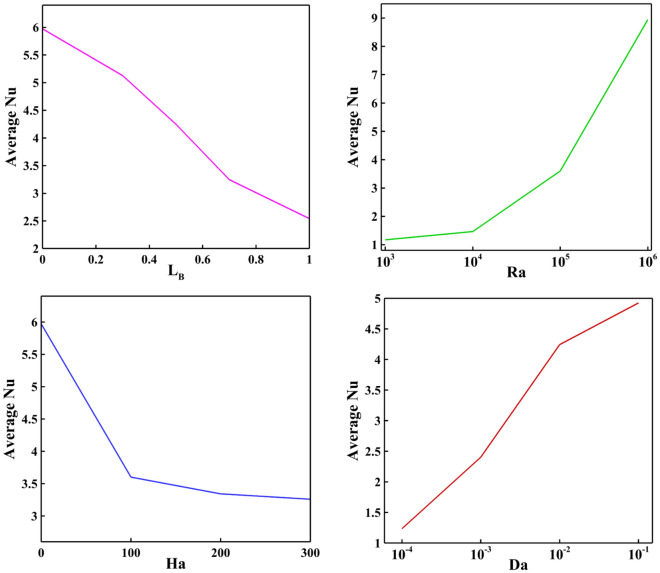
Impact of magnetic field length, Rayleigh, Hartmann, and Darcy numbers on Average Nusselt with Ra = 10^5^, Da = 0.01, ϵ_p_ = 0.9, Ha = 50, L_B_ = 0.5, $k {\prime}$= 2.

**Figure 16:**
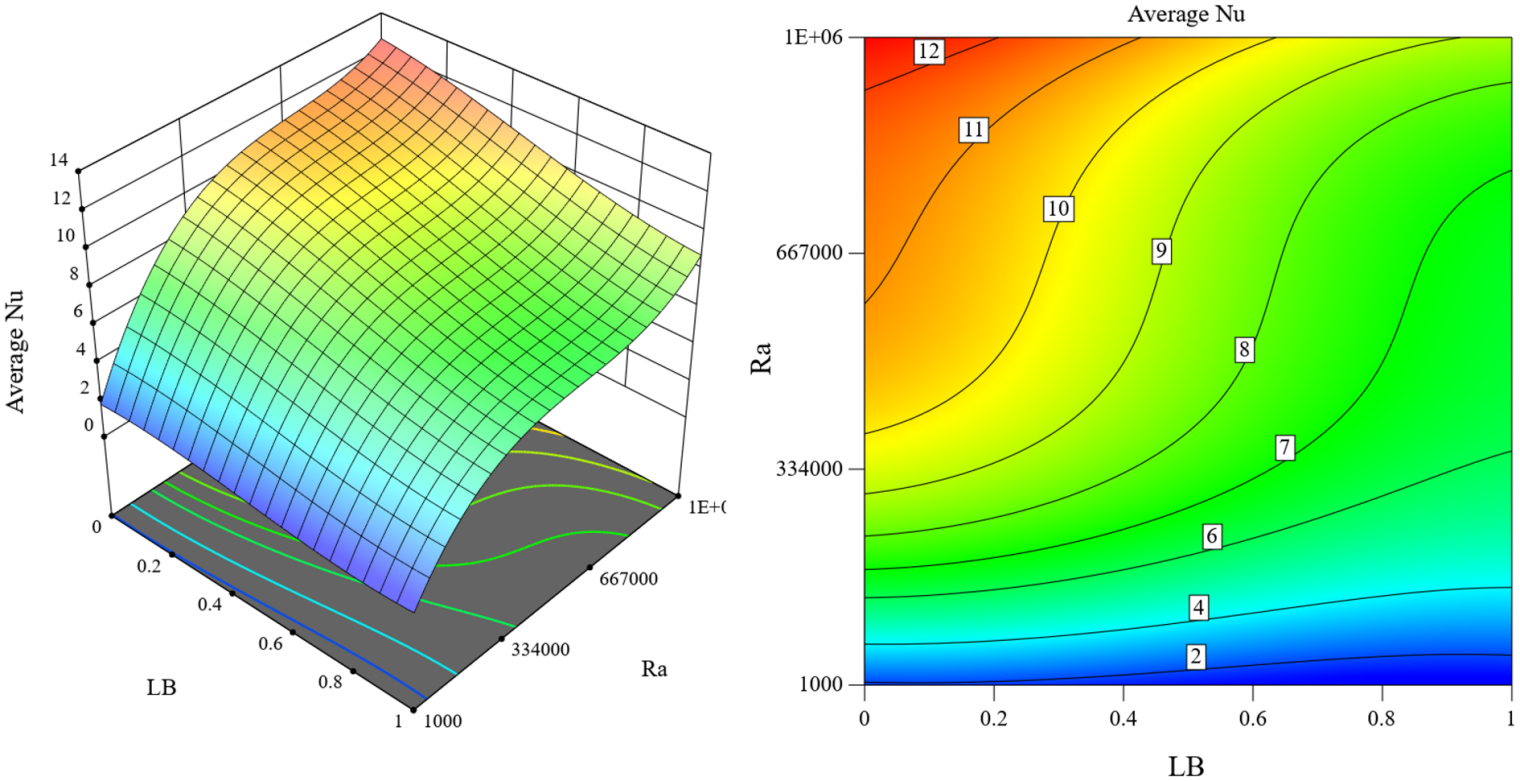
Simultaneous effect of magnetic field length and Rayleigh number on average Nusselt number with Da = 0.01, ϵ_p_ = 0.9, Ha = 50, $k$ = 2.

**Figure 17:**
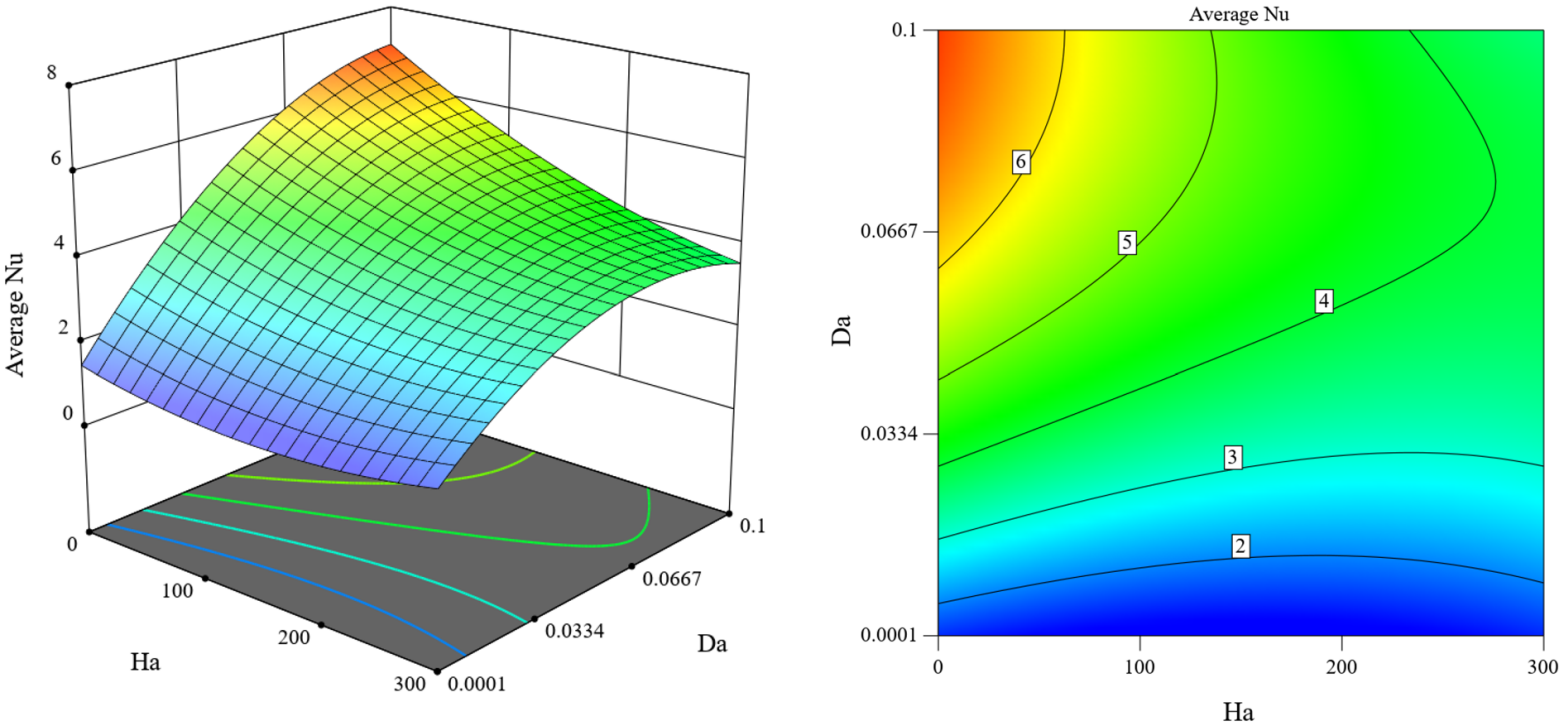
Simultaneous impact of Hartmann and Darcy numbers on average Nusselt number with Ra = 10^5^, ϵ_p_ = 0.9, L_B_ = 0.5, $k$ = 2.

Average Nusselt is influenced by magnetic field length, Rayleigh, Hartmann, and Darcy number, as shown in Fig. 15. The average Nusselt is proportional to the Rayleigh and Darcy numbers and increases with the increase of Rayleigh and Darcy. At the same time, it has an inverse relationship with the Hartmann number and the length of the magnetic field. Figures 16 and 17 show the simultaneous effect of magnetic field length, Rayleigh number, and Hartmann and Darcy numbers on the average Nusselt. The average Nusselt number reached its highest point when there was no magnetic field, and the Rayleigh and Darcy numbers were high.

In summary, the set of average Nusselt number and maximum psi value is given in Table 3.

**Table 3:** The values of average Nusselt number and maximum psi with Ra = 10^5^, Da = 0.01, ϵ_p_ = 0.9, Ha = 50, L_B_ = 0.5, $k{\prime}$ = 2.

L_B_	0.3	0.5	0.7
$\overline{\text{Nu} }$	5.127	4.245	3.247
${\left|\Psi \right|}_{Max}$	8	7	5.28
Ra	10^4^	10^5^	10^6^
$\overline{\text{Nu} }$	1.465	3.6	8.944
${\left|\Psi \right|}_{Max}$	1.7	6	14
Ha	0	100	200
$\overline{\text{Nu} }$	5.973	3.6	3.342
${\left|\Psi \right|}_{Max}$	9	6	5.5
Da	0.0001	0.001	0.01
$\overline{\text{Nu} }$	1.234	2.404	4.245
${\left|\Psi \right|}_{Max}$	0.65	3.4	7
$\text{k}{\prime}$	0	1	2
$\overline{\text{Nu} }$	3.978	4.093	4.245
${\left|\Psi \right|}_{Max}$	6.91	6.93	7

## Conclusion

This paper presents the results of a numerical study on natural convection in a square wavy cavity in the presence of a partial magnetic field under effective factors such as magnetic field length (L_B_), Rayleigh number (Ra), Hartmann number (Ha), Darcy number (Da) and wave number ($k{\prime}$). The effective parameters were investigated in L_B_ = 0.3–0.7, Ra = 10^4^–10^6^, Da = 0.0001–0.01, $k{\prime}$ = 0–3 intervals. In addition, the distribution of temperature, average Nusselt, streamlines, and isotherm lines were shown for the cavity. The most important results obtained are as follows:

1. As the magnetic field’s length increases, heat transfer decreases. As the length of the magnetic field increases from 0.3 to 0.7, the Nusselt number decreases by 36.6%, and the value of maximum psi decreases by 34%.
2. As the Rayleigh number increases, the Nusselt number also increases. In fact, with the increase of the Rayleigh number, the buoyancy force increases, and the buoyancy force increases the heat transfer and fluid velocity. As the Rayleigh number increases from 10^4^ to 10^6^, the Nusselt number increases more than five times, and the value of maximum psi increases more than seven times.
3. In the lower Rayleigh, the second vortex is formed due to the dominance of the Lorentz force over the buoyancy force.
4. The effect of Lorentz force on fluid flow and heat transfer increases at a high Rayleigh number.
5. Nusselt number decreases with increasing Hartmann number. As the Hartmann number increases, the Lorentz force increases. Lorentz force reduces heat transfer and fluid velocity. As the Hartmann number increases from 0 to 200, the Nusselt number decreases by 44%, and the value of maximum psi decreases by 38.8%.
6. By increasing the permeability in the porous medium, heat transfer is improved. As the Darcy number increases from 0.0001 to 0.01, the Nusselt number increases more than two times, and the value of maximum psi increases more than nine times.
7. At low Darcy numbers, the magnetic field becomes ineffective.
8. As the wave number of the right wall increases, the Nusselt number increases due to the increase of the heat transfer area. As the wave number increases from 0 to 2, the Nusselt number increases by 6.71%, and the value of maximum psi increases by 1.3%.

## Data Availability

The datasets used and/or analysed during the current study available from the corresponding author on reasonable request.

## List of symbols

Pr: Prandtl number
Ra: Rayleigh number
Ha: Hartmann number
Da: Darcy number
${\text{c}}_{\text{p}}$: Specific heat, J.kg^-1^.K^-1^
$${\upepsilon }_{\text{p}}$$: Porosity
$$k$$: Thermal conductivity, W.m^−1^.K^−1^
$$K$$: Permeability,m^2^
$$\text{T}$$: Temperature, K
$$\text{g}$$: Gravitational acceleration
$\text{L}$: Length of the cavity, m
$\text{H}$: Height of the cavity, m
$$k{\prime}$$: Wave number
${\text{L}}_{\text{B}}$: Magnetic field length
$\overline{\text{Nu} }$: Average Nusselt number
$$\text{u},\text{v}$$: Velocity, m.s^-1^
$${\text{u}}^{{{\prime}}},{\text{v}}^{{{\prime}}}$$: Dimensionless velocity
t: Time, s
$${\text{t}}^{{{\prime}}}$$: Dimensionless time
$$\text{p}$$: Pressure, pa
$${\text{p}}^{{{\prime}}}$$: Dimensionless pressure
${\text{B}}_{0}$: Magnitude of the magnetic field, N.A^-1^.m^2^
$$\text{x},\text{y}$$: Cartesian coordinates,m
${\text{x}}^{{{\prime}}},{\text{y}}^{{{\prime}}}$: Dimensionless cartesian coordinates

## Greek letters

$$\uptheta $$: Dimensionless temperature
$$\uprho $$: Density, kg.m^-3^
$\upmu$: Dynamic viscosity, Pa.S
$\upbeta$: Thermal expansion coefficient, K^-1^
$${\alpha }$$: Thermal diffusivity, m^2^.s
$$\omega $$: Vorticity
$\psi$: Dimensionless stream function

## Subscripts

$$e$$: Effective
$$f$$: Fluid
*s*: Solid
*h*: Hot
c: Cold

## References

[1] Baïri, A., Esther Z. P., & García De María, J. M.. A review on natural convection in enclosures for engineering applications. The particular case of the parallelogrammic diode cavity. *Appl. Thermal Eng. 63*(1), 304–322 (2014).

[2] Asghar, Z., Muhammad, W. S. K., Amjad, A. P., Mustafa, M. R., Sankaralingam, L., & Mohammad, I. A. On non-Newtonian fluid flow generated via complex metachronal waves of cilia with magnetic, hall, and porous effects. *Phys. Fluids* **35**(9) (2023).

[3] Umavathi, J. C., & Mahanthesh, B. Study of multilayer flow of two immiscible nanofluids in a duct with viscous dissipation. *Phys. Fluids* **35**(9) (2023).

[4] Parmar, D., Rathish Kumar, B. V., Krishna Murthy, S. V., & Sumant, K. Numerical study of entropy generation in magneto-convective flow of nanofluid in porous enclosure using fractional order non-Darcian model. *Phys. Fluids* **35**(9) (2023).

[5] Rath, C., & Anita, N. A numerical study on MHD Cu-Al2O3/H2O hybrid nanofluid with Hall current and cross-diffusion effect. *Phys. Fluids* **35**(10) (2023).

[6] Rahimi, A., Ali, D. S., Abbas K., & Emad, H. M. A comprehensive review on natural convection flow and heat transfer: the most practical geometries for engineering applications. *Int. J. Numer. Methods Heat Fluid Flow* **29**(3), 834–877 (2019).

[7] Sreedevi, P., & Sudarsana Reddy, P. Effect of magnetic field and thermal radiation on natural convection in a square cavity filled with TiO2 nanoparticles using Tiwari-Das nanofluid model. *Alexandria Eng. J.* **61**(2), 1529–1541 (2022).

[8] Dogonchi AS, Armaghani T, Chamkha Ali J, Ganji DD (2019). Natural convection analysis in a cavity with an inclined elliptical heater subject to shape factor of nanoparticles and magnetic field. Arabian J. Sci. Eng..

[9] Li, Z., Ahmed, K. H., Obai, Y., Masoud, A., & Shizhe, F. Natural convection and entropy generation of a nanofluid around a circular baffle inside an inclined square cavity under thermal radiation and magnetic field effects. *Int. Commun. Heat Mass Transf.* **116**, 104650 (2020).

[10] Sattar Dogonchi A, Tayebi T, Karimi N, Chamkha AJ, Alhumade H (2021). Thermal-natural convection and entropy production behavior of hybrid nanoliquid flow under the effects of magnetic field through a porous wavy cavity embodies three circular cylinders. J. Taiwan Inst. Chem. Eng..

[11] Jalili P, Afifi MD, Jalili B, Mirzaei AM, Ganji DD (2023). Numerical study and comparison of two-dimensional ferrofluid flow in semi-porous channel under magnetic field. Int. J. Eng..

[12] Mirzaei A, Jalili P, Afifi MD, Jalili B, Ganji DD (2023). Convection heat transfer of MHD fluid flow in the circular cavity with various obstacles: Finite element approach. Int. J. Thermofluids.

[13] Jalili, P., Mohammad, D. A., Amirmohammad, M., Bahram, J., & Davood, D.G. Study of ferrofluid flow with Lorentz force in the porous channel in the presence of transversely magnetic field. *Int. J. Eng.* (2023).

[14] Dehghan, A., *et al*. The effects of thermal radiation, thermal conductivity, and variable viscosity on ferrofluid in porous medium under magnetic field. *World J. Eng*. (2024).

[15] Jalili P (2023). Analytical and numerical investigation of thermal distribution for hybrid nanofluid through an oblique artery with mild stenosis. SN Appl. Sci..

[16] Jalili, P., *et al*. Thermal analysis of transverse fluid flow in a gradient porous media with the exponentially boundary conditions. *Mod. Phys. Lett. B* 2350229 (2023).

[17] Bahmani M (2024). Effect of variations hollow of octagon porous media on heat and mass transfer. Int. J. Thermofluids.

[18] Mahmoudi A (2019). A scale analysis for natural convection in a porous media in the presence of a magnetic field. J. Taiwan Inst. Chem. Eng..

[19] Izadi M, Rasul M, Amin AD, Hasan S (2019). Natural convection of a magnetizable hybrid nanofluid inside a porous enclosure subjected to two variable magnetic fields. Int. J. Mech. Sci..

[20] Massoudi, M. D., Mohamed, B. B. H., & Mohammed, A. A. Free convection and thermal radiation of nanofluid inside nonagon inclined cavity containing a porous medium influenced by magnetic field with variable direction in the presence of uniform heat generation/absorption. *Int. J. Numer. Methods Heat Fluid Flow* **31**(3), 933–958 (2021).

[21] Qi C, Tang J, Ding Zi, Yan Y, Guo L, Ma Y (2019). Effects of rotation angle and metal foam on natural convection of nanofluids in a cavity under an adjustable magnetic field. Int. Commun. Heat Mass Transfer.

[22] Hussein AK (2022). Natural convection in F-shaped cavity filled with Ag-water non-Newtonian nanofluid saturated with a porous medium and subjected to a horizontal periodic magnetic field. Korean J. Chem. Eng..

[23] Izadi M (2019). LTNE modeling of Magneto-Ferro natural convection inside a porous enclosure exposed to nonuniform magnetic field. Phys. A Stat. Mech. Appl..

[24] Hashemi H, Zafar N, Mehryan SAM (2017). Cu-water micropolar nanofluid natural convection within a porous enclosure with heat generation. J. Mol. Liquids.

[25] Li F, Yahya AR, Alibek I, Mahmoud MS, Xiaoduo O, Li Z (2021). "Free convection simulation of hybrid nanomaterial in permeable cavity with inclusion of magnetic force. J. Mol. Liquids.

[26] Nong H (2021). Numerical modeling for steady-state nanofluid free convection involving radiation through a wavy cavity with Lorentz forces. J. Mol. Liquids.

[27] Gibanov NS, Sheremet MA, Pop I (2016). Natural convection of micropolar fluid in a wavy differentially heated cavity. J. Mol. Liq..

[28] Mehmood K, Hussain S, Sagheer M (2017). Numerical simulation of MHD mixed convection in alumina–water nanofluid filled square porous cavity using KKL model: Effects of nonlinear thermal radiation and inclined magnetic field. J. Mol. Liq..

[29] Toosi MH, Majid S (2017). Two-phase mixture numerical simulation of natural convection of nanofluid flow in a cavity partially filled with porous media to enhance heat transfer. J. Mol. Liquids.

[30] Geridonmez, Pekmen B, Oztop HF (2020). MHD natural convection in a cavity in the presence of cross partial magnetic fields and Al2O3-water nanofluid. Comput. Math. Appl..

[31] Geridonmez G, Pekmen B, Oztop HF (2019). Natural convection in a cavity filled with porous medium under the effect of a partial magnetic field. Int. J. Mech. Sci..

[32] Pekmen Geridonmez B, Oztop HF (2020). Natural convection in a cavity under partial magnetic field applied from different corners. Int. Commun. Heat Mass Trans..

[33] Selimefendigil F, Öztop HF (2020). Effects of conductive curved partition and magnetic field on natural convection and entropy generation in an inclined cavity filled with nanofluid. Phys. A.

[34] Hussain S, Shoeibi S, Armaghani T (2021). Impact of magnetic field and entropy generation of Casson fluid on double diffusive natural convection in staggered cavity. Int. Commun. Heat Mass Trans..

[35] Li Z (2019). Simulation of natural convection of Fe 3 O 4-water ferrofluid in a circular porous cavity in the presence of a magnetic field. Eur. Phys. J. Plus.

[36] Afrand M (2020). Free convection and entropy generation of a nanofluid in a tilted triangular cavity exposed to a magnetic field with sinusoidal wall temperature distribution considering radiation effects. Int. Commun. Heat Mass Trans..

[37] Saleem, S., *et al*. Steady laminar natural convection of nanofluid under the impact of magnetic field on two-dimensional cavity with radiation. *AIP Adv.* **9**(6) (2019).

[38] Belhaj S, Ben-Beya B (2022). Thermal performance analysis of hybrid nanofluid natural convection in a square cavity containing an elliptical obstacle under variable magnetic field. Int. J. Numer. Meth. Heat Fluid Flow.

[39] Rahman MM, Pop I, Saghir MZ (2019). Steady free convection flow within a titled nanofluid saturated porous cavity in the presence of a sloping magnetic field energized by an exothermic chemical reaction administered by Arrhenius kinetics. Int. J. Heat Mass Transf..

[40] Mourad A, Aissa A, Mebarek-Oudina F, Al-Kouz W, Sahnoun M (2021). Natural convection of nanoliquid from elliptic cylinder in wavy enclosure under the effect of uniform magnetic field: Numerical investigation. Eur. Phys. J. Plus.

[41] Abderrahmane A (2023). Investigation of the free convection of nanofluid flow in a wavy porous enclosure subjected to a magnetic field using the Galerkin finite element method. J. Magnetism Mag. Mater..

[42] Nabwey HA (2023). Radiative effects on unsteady MHD natural convection flow in an inclined wavy porous cavity using hybrid nanofluid containing a square obstacle. Alexandria Eng. J..

[43] Salehi M, Afshar SR, Ali R, Chamkha AJ (2023). Numerical investigation of TiO2-water nanofluid heat transfer in a porous wavy circular chamber with a ┴-shaped heater under magnetic field. Case Stud. Thermal Eng..

[44] Abderrahmane A (2023). Second law analysis of a 3D magnetic buoyancy-driven flow of hybrid nanofluid inside a wavy cubical cavity partially filled with porous layer and non-Newtonian layer. Annals Nuclear Energy.

[45] Alqaed S (2023). Entropy generation of the laminar and mixed flow of alumina/water nanofluid flow in a two-dimensional rectangular enclosure affected by a magnetic field using the lattice Boltzmann method Eng. Anal. Boundary Elements.

[46] Jelodari I, Nikseresht AH (2018). Effects of Lorentz force and induced electrical field on the thermal performance of a magnetic nanofluid-filled cubic cavity. J. Mol. Liq..

[47] Ahrar AJ, Mohammad HD (2016). Lattice Boltzmann simulation of a Cu-water nanofluid filled cavity in order to investigate the influence of volume fraction and magnetic field specifications on flow and heat transfer. J. Mol. Liquids.

[48] Alsabery AI, Mohebbi R, Chamkha AJ, Hashim I (2019). Effect of local thermal non-equilibrium model on natural convection in a nanofluid-filled wavy-walled porous cavity containing inner solid cylinder. Chem. Eng. Sci..

[49] Alsabery AI, Tayebi T, Chamkha AJ, Hashim I (2018). Effect of rotating solid cylinder on entropy generation and convective heat transfer in a wavy porous cavity heated from below. Int. Commun. Heat Mass Trans..

[50] Alsabery AI, Abosinnee AS, Al-Hadraawy SK, Ismael MA, Fteiti MA, Hashim I, Sheremet M, Ghalambaz M, Chamkha AJ (2023). Convection heat transfer in enclosures with inner bodies: A review on single and two-phase nanofluid models. Renew. Sustain. Energy Rev..

